# Supplementary material for: Iterative improvement in the automatic modular design of robot swarms
Source: PeerJ Comput Sci. 2020 Dec 7;6:e322. doi: 10.7717/peerj-cs.322 (PMC7924708; doi:10.7717/peerj-cs.322)
Supplement: Supplemental Information 3 [file peerj-cs-06-322-s003.zip › argos3/doc/api/standalone/a00605_source.html]

ARGoS: plugins/simulator/physics\_engines/dynamics2d/dynamics2d\_differentialsteering\_control.h Source File


- Main Page
- Related Pages
- Namespaces
- Classes
- Files

- File List
- File Members

# plugins/simulator/physics\_engines/dynamics2d/dynamics2d\_differentialsteering\_control.h

Go to the documentation of this file.

```
00001 
00007 #ifndef DYNAMICS2D_DIFFERENTIALSTEERING_CONTROL_H
00008 #define DYNAMICS2D_DIFFERENTIALSTEERING_CONTROL_H
00009 
00010 namespace argos {
00011    class CDynamics2DEngine;
00012 }
00013 
00014 #include <argos3/plugins/simulator/physics_engines/dynamics2d/dynamics2d_velocity_control.h>
00015 #include <argos3/core/utility/math/vector2.h>
00016 
00017 namespace argos {
00018 
00019    class CDynamics2DDifferentialSteeringControl : public CDynamics2DVelocityControl {
00020 
00021    public:
00022       
00023       CDynamics2DDifferentialSteeringControl(CDynamics2DEngine& c_engine,
00024                                              Real f_max_force,
00025                                              Real f_max_torque,
00026                                              Real f_interwheel_distance);
00027 
00028       virtual ~CDynamics2DDifferentialSteeringControl() {}
00029 
00030       void SetWheelVelocity(Real f_left_wheel,
00031                             Real f_right_wheel);
00032 
00033       inline Real GetInterwheelDistance() const {
00034          return m_fInterwheelDistance;
00035       }
00036 
00037    private:
00038 
00039       Real m_fInterwheelDistance;
00040 
00041    };
00042 
00043 }
00044 
00045 #endif
```

---

Generated on 10 Jul 2018 for ARGoS by 
 1.6.1
